# Drug Abuse among University Students of Rafsanjan, Iran

**Published:** 2014

**Authors:** Omid Rezahosseini, Ali Roohbakhsh, Vahid Tavakolian, Sepideh Assar

**Affiliations:** 1 Medical Student, Students Research Committee, School of Medicine, Ranfsanjan University of Medical Sciences, Rafsanjan, Iran.; 2 Assistant Professor, Physiology-Pharmacology Research Center, Rafsanjan University of Medical Sciences, Rafsanjan, Iran.; 3 Dental Student, Students Research Committee, School of Dentistry, Ranfsanjan University of Medical Sciences, Rafsanjan, Iran.

**Keywords:** Cigarette, Drug Abuse, Methylphenidate, Student, Tobacco

## Abstract

**Objective:** The present study aimed to determine the frequency of drug abused in a sample of university students in Rafsanjan, Iran.

**Methods:** In this cross-sectional study, 1,260 students volunteered (311 males and 949 females) with mean age of 21.35 years. Data were collected by a self-administrated questionnaire regarding drugs abuse and demographic information.

**Results: **Benzodiazepines were the most common abused drugs which were reported in 94 students (7.4%). Other agents studied were cigarette and tobacco (159, cases 12.6%), alcoholic drinks (60 cases, 4.7%), and opiates (42 cases, 3.3%). Forty-three students (3.4%) had used methylphenidate in the last 6 months. Of this, 39 (90.6%) experienced insomnia. All students who abused methylphenidate indicated that the reason for this behavior was to raise alertness and conscious levels.

**Conclusion:** The pattern of drug abuse among the students here seems similar to other reports from Iranian universities.

## Introduction

Abusing drugs such as alcohol, narcotics, analgesics and ecstasy is getting more common among young adults. Documents show that 8.3% of those who are more than 12 years have abused at least one drug in the last month (1).

The most common age range of drug abusers in the world is 18 to 25 years (2). Lots of students are at these ages and drug abuse rise has been proved in them. Among illicit abused drugs, marijuana with consumption of 3.8% is the most common abused drug in the world (3). Race, gender, social class, religion and government rules, availability (2), persuading or pressure of friends, education and job stress and curiosity are the most common reasons of drug abuse among general population (4).

Drug abuse is seen in Iran despite this fact that law and religion prohibit it and it is not acceptable culturally among Iranian families (5). According to reports, 0.3-0.9% of drug abusers are students (6) and cigarette, opium, cannabis and alcohol are the most common drugs abused (7).

In a study in Tabriz, Iran among high school students, 10.1% had at least once experienced alcohol consumption and 2.2% experienced drug abuse (5). In a survey among students in Tehran -capital of Iran- frequency of using Hookah was 34%, cigarette 24%, alcoholic drinks 17%, opium 2.3%, cannabis 2.2%, and ecstasy was 0.7% (8).

Amphetamines like methylphenidate are popular among students and are known as “study drug” (9). Commonly, these drugs are used for concentration and consciousness increase (10). The frequency of abusing such agents is estimated to be 0.5-35.3% (10, 11). There is little information about methylphenidate abuse among Iranian students and its abuse has complications such as dependency, drug tolerance and drug side effects.

## Materials and Methods

In this cross-sectional study carried out during winter 2008, all the students (6,500 students) in three universities including Rafsanjan University of Medical Sciences, Valieasr University and Rafsanjan Branch of Islamic Azad University were announced and invited to participate in this study by filling out an anonymous self-administered questionnaire. A total of 1260 volunteers participated in this study (participation rate of 19.3%) (1). For data collection, we used a self-administrated questionnaire in Persian, which had two parts i.e. demographic information and study-related questions. Demographic information included gender, marital status, age, year of university entrance, and level of parents’ education. Study-related questions were about drugs abused including benzodiazepines, anti-depressants, antipsychotics, anticonvulsants, Propranolol, cigarette and tobacco, alcoholic drinks, ecstasy, opium and its derivates, anabolics and specially methylphenidate.

The students were asked about the methylphenidate by the following questions:

- If they know this drug
- Use of methylphenidate in the last 6 months
- Knowledge about its side effects
- The person who prescribed the drug
- Reason of usage
- Experienced side effects by using this drug.

The questionnaire was validated by experts (two pharmacologists, a psychiatrist, and two methodologists) and the Cronbach’s alpha for reliability obtained 0.87. Then the questionnaire were coded and passed to the volunteers. A medical student was on call to answer the questions probably asked by the study subjects. Personal information that could reveal students’ identity was not required. This was to ensure confidentiality and to encourage more participation and expression of opinions. Students were instructed to return the filled questionnaires to the author through the special boxes placed in the universities.

Collected data were analyzed by the SPSS for Windows 17.0 (SPSS Inc., Chicago, IL, USA) and we used descriptive indices and chi-square test to analyze the data. p < 0.05 was statistically considered as a significant level. Research Committee of Rafsanjan University of Medical Sciences proved the study design and its methods.

## Results

Among 1,260 participants in the study, 311 (24.7%) were males and 949 (75.3%) were females with a mean (±SD) age of 21.35 (±2.73) years. Regarding marital status, 1,102 (87.5%) students were single, 143 (11.4%) married, and 15 (1.1%) were divorced. In terms of university level, 122 (9.7%) of the participants were students of pre-bachelor degree, 965 (76.6%) bachelor degree, 26 (2.1%) masters and 147 (11.6%) were students at PhD, DDS or MD levels. Among CNS-affecting drugs (central nervous system), benzodiazepines were the most frequent abused agent reported in 94 students (7.4%). The frequency of using benzodiazepines among males was 38 (12.2%) students and among females 56 (5.9) students (p = 0.002). Frequency of students who reported cigarette and tobacco usage was 159 (12.6%), alcoholic drinks 60 (4.7), and opium and its derivatives was 42 (3.3%) students.

Frequency and comparison of drugs abused based on gender, marital status, and residential place are presented in table 1. 

In terms of methylphenidate, 262 (20.8%) students already knew the drug, 43 (3.4%) had used it in the last 6 months and 188 (14.9%) were aware of its side effects. Frequency of methylphenidate usage was 20 (6.4%) in male and 23 (2.4%) in female students (p = 0.024). The prescription of methylphenidate in 14 (32.4%) students was by a physician and in 8 students (19.2%) had a self-administrated consumption (p < 0.0001). Table 2 illustrates the frequency of people who prescribed or advised the usage of methylphenidate based on gender, marital status, and residential place. The reasons of methylphenidate usage in order of decrease were increasing alertness (43 subjects, 100%), studying better (31 subjects, 72%), increasing concentration (19 subjects, 44.1%), mood elevation (18 subjects, 41.8%), and curiosity (8 subjects, 18.6%), respectively. About methylphenidate users, the experienced side effects were insomnia (39 subjects, 90.6%), palpitation (18 subjects, 41.8%), headache (14 subjects, 32.5%), lightheadedness (13 subjects, 30.2%), anxiety (13 subjects, 30.2%), flashing (13 subjects, 30.2%), nausea and vomiting (9 cases, 20.9%), anorexia (9cases, 20.9%), skin complications (3 subjects, 6.9%), visual disturbances (3 subjects, 6.9%), and convulsion (one subject, 2.3%).

## Discussion

The results of this study showed that benzodiazepines whose abuse was reported in 94 students (7.4%) were the most common abused drugs. Unfortunately, there is not any similar data of abusing this group of drugs among Iranian students for comparison. Due to gender, drug abuse was more common in male students than in females, which was statistically significant (p < 0.002). In the students with rental houses, the abuse of cigarette, tobacco and alcohol was more common. This might be due to being far away from parental control. This finding is in agreement with previous reports (8-12). But frequency of opium abuse was more common in students who live with their family (p < 0.0001). It might be due to imitating family behavior. However, this behavior needs to be studied further by other studies. 

**Table 1:** Frequency and comparison of abused drugs based on gender, marital status, and residential place

**Demographic characteristics** **Drug names**	**Gender**		**Marital status**				**Place of residency**				**Consumption in total**
	**Male** **(n=311)**	**Female** **(n=949)**	**Single** **(n = 1102)**	**Married** **(n = 143)**	**Divorced** **(n = 15)**		**With family** **(n = 144)**	**Dormitory** **(n = 1053)**		**Rental house** **(n = 63)**	
**Benzodiazepines** **n’ (%)**	38 (12.2)	56 (5.9)	66 (5.9)	22 (15.3)	6 (40.0)		19 (13.1)	70 (6.6)		5 (7.9)	94 (7.4)
**P-value**	0.002		< 0.0001				0.029				-
**Anti-Depressant** **n’ (%)**	22 (7.0)	48 (5.0)	54 (4.9)	15 (10.4)	1 (6.6)		7 (4.8)	59 (5.6)		4 (6.3)	70 (5.5)
**P-value**	0.386		0.036				0.91				-
**Anti-Psychotic** **n’ (%)**	24 (7.7)	46 (4.8)	49 (4.5)	17 (11.6)	4 (26.6)		12 (8.3)	51 (4.8)		7 (11.1)	70 (5.5)
**P-value**	0.113		<0.0001				0.034				-
**Anti-convulsant** **n’ (%)**	10 (3.2)	13 (1.3)	19 (1.7)	4 (2.7)	0 (0.0)		5 (3.4)	16 (1.5)		2 (3.1)	23 (1.8)
**P-value**	0.084		0.528				0. 147				-
**Propranolol** **n’ (%)**	10 (3.2)	50 (5.2)	54 (4.9)	6 (4.1)	0 (0.0)		1 (0.6)	56 (5.3)		3 (4.7)	60 (4.7)
**P-value**	0.07		0.708				0.708				-
**Cigarette and Tobacco** **n’ (%)**	110 (35.3)	49 (5.1)	124 (11.2)	26 (18.1)	9 (60.0)		34 (23.6)	106 (10.0)		19 (30.1)	159 (12.6)
**P-value**	< 0.0001		<0.0001				< 0.0001				-
**Alcoholic drinks** **n’ (%)**	44 (14.1)	16 (1.6)	43 (3.9)	11 (7.6)	6 (40.0)		13 (9.0)	39 (3.7)		8 (12.6)	60 (4.7)
**P-value**	< 0.0001		<0.0001				< 0.0001				-
**Ecstasy drugs n’ (%)**	5 (1.6)	18 (1.8)	18 (1.6)	4 (2.7)	1 (6.6)		2 (1.3)	19 (1.8)		19 (1.8)	23 (1.8)
**P-value**	< 0.0001		0.187				0.676				-
**Opium and derivatives** **n’ (%)**	25 (8.0)	17 (1.7)	29 (2.6)	9 (6.2)	4 (26.6)		13 (9.0)	28 (2.6)		1 (1.5)	42 (3.3)
**P-value**	< 0.0001		< 0.0001				< 0.0001				-
**Anabolics** **n’ (%)**	1 (0.3)	7 (0.7)	6 (0.5)	1 (0.6)		1 (6.6)	0 (0.00)		7 (0.6)	1 (1.5)	8 (0.6)
**P-value**	0.999		0.007				0.4				-

n’/n×100 = frequency percent in subgroup; n’/n×*100 = frequency percent in total population

**Table 2:** Frequency of people who prescribed or advised the use of methylphenidate based on gender, marital status, and residential place

**Demographic characteristics** **Prescriber**	**Gender**		**Marital status**			**Place of residency**		
	**Male** **(n =311) n (%)**	**Female** **(n =949)** **n (%)**	**Single** **(N=1102) (%)n**	**Married** **(n = 143)** **n (%)**	**Divorced** **(n = 15)** **n (%)**	**With family** **(n = 144)** **n (%)**	**Dormitory** **(n = 1053)** **n (%)**	**Rental house** **(n = 63)** **n (%)**
**Physician**	160 (51.4)	263 (27.7)	314 (28.5)	74 (51.7)	3 (20.0)	79(54.9)	298 (28.4)	39 (62.0)
**Pharmacist**	019 (6.1)	014 (1.4)	024 (2.2)	03 (2.1)	3(20.0)	05 (3.5)	023 (2.2)	04 (6.4)
**Friends**	047 (15.1)	039 (4.1)	071 (6.4)	08 (5.6)	0.0	18 (12.5)	056 (5.3)	04 (6.4)
**Self-administrated**	075 (24.1)	167 (17.5)	190 (17.2)	34 (23.8)	9 (60.0)	42 29.1)	195 (18.5)	08 (12.6)
**Others**	10 (3.3)	466 (49.3)	503 (45.7)	24 (16.8)	0.0	0.0	481 (45.6)	08 (12.6)

n/n×100=frequency percent of subgroup

Abusing these drugs was more common in divorced students as it was expected. Furthermore, abusing benzodiazepines and antipsychotics were more common in this group as well. Drug abuse in divorced people might be the result of stress of divorcing, and the other guess is that abusing the drugs and having no moral equilibrium might be the reason of divorcing.

Regarding the records, prevalence of drug abuse among students in Iran has become three folds more common when compared to Iran’s population growth in the last 20 years (13). This trend has occurred due to different reasons and lack of knowledge about side effects of abused drugs which might be an explanation for this rising figure (4). The interesting point is that 38.2% of drug abuser in Iran, abuse opium just for enjoying its euphoric effects and 19.4% for its narcotic effects to reduce physical pain. Abusing opium is accompanied by abusing other illicit drugs as well (7).

In different reports of Saudi Arabia, drug and alcohol abuse among men of 15-30 years has been mentioned as a public health issue. This problem has been surveyed in Lebanon, Brazil and Turkey with similar results (1, 3).

In the present study, methylphenidate abuse was observed in 43 students (3.4%) in the last 6 months. This figure was less than the former (6.5% to 35.5%) (10, 11). Male students in comparison to females were more familiar with this drug and had more information about its side effects and they used it more than females in the last 6 months. This was in agreement with previous studies (9). In other researches, a statistical significant association was shown between methylphenidate abuses and abusing other drugs; however, we did not find such an association in our data (13, 14).

Insomnia and effects like headache which were common side effects of methylphenidate taking in exam period (which is the most methylphenidate abuse time) and would make irreversible effects on students’ education.

## Conclusion

Regarding the results of this study, life stressors such as divorcing, living far from family and living in rental houses without family controlling role, could be related to drug abuse. Curiosity of young adults is also another important reason. Increasing the training programs can answer many questions asked by the youth in this case. Therefore, more accurate studies of drug abuse condition in other universities and exact programming for prevention of drug abuse are necessary.

## Authors' contributions

AR and OR conceived and designed the evaluation and helped to draft the manuscript. JA participated in designing the evaluation and performed parts of the statistical analysis. OR and AR re-evaluated the clinical data, revised the manuscript and performed the statistical analysis and revised the manuscript. VT, OR and AR collected the clinical data, interpreted them and revised the manuscript. All authors read and approved the final manuscript.

## Footenote

**Declaration of interest:** None.
